# Trajectory of cognitive decline before and after incident arrhythmias in older adults: A 16‐year population‐based longitudinal cohort study

**DOI:** 10.1002/alz.70260

**Published:** 2025-05-19

**Authors:** Haibin Li, Frank Qian, Jianian Hua, Man Wang, Jiao Wang, Xinye Zou, Zhiyuan Wu, Xia Li, Xiuhua Guo, Wuxiang Xie

**Affiliations:** ^1^ Department of Cardiac Surgery Heart Center Beijing Chaoyang Hospital Capital Medical University Beijing China; ^2^ Section of Cardiovascular Medicine Boston Medical Center and Boston University Chobanian & Avedisian School of Medicine Boston Massachusetts USA; ^3^ Department of Neurology The First Affiliated Hospital of Soochow University Suzhou China; ^4^ Department of Cardiology China‐Japan Friendship Hospital Beijing China; ^5^ Center of Gerontology and Geriatrics National Clinical Research Center for Geriatrics West China Hospital Sichuan University Chengdu China; ^6^ Department of Neurobiology, Care Sciences and Society Karolinska Institutet Stockholm Sweden; ^7^ Cambridge Public Health University of Cambridge Cambridge UK; ^8^ Department of Nutrition Harvard T.H. Chan School of Public Health Boston Massachusetts USA; ^9^ Department of Mathematics and Statistics La Trobe University Melbourne Victoria Australia; ^10^ Department of Epidemiology and Health Statistics Capital Medical University School of Public Health Beijing China; ^11^ Peking University Clinical Research Institute Peking University First Hospital Beijing China

**Keywords:** arrhythmias, cognitive decline, cohort study, trajectory

## Abstract

**INTRODUCTION:**

The temporal pattern of cognitive decline before and after incident arrhythmias remains largely unknown.

**METHODS:**

This population‐based cohort study included 6,494 participants (mean age: 62.9 ± 9.4 years) from the English Longitudinal Study of Ageing. Participants underwent a cognitive assessments at baseline and at least one subsequent time point.

**RESULTS:**

During a median follow‐up of 12 years, 628 individuals were diagnosed with incident arrhythmias. Overall, the annual rate of cognitive decline before arrhythmia diagnosis among individuals who experienced incident arrhythmias was similar to that of participants who were free of arrhythmias throughout follow‐up. No short‐term cognitive decline was observed in participants with arrhythmia diagnosis after the event. Individuals with incident arrhythmias demonstrated a faster decline in global cognition (−0.042 standard deviation/year; 95% confidence interval: −0.065 to −0.019) over the years after arrhythmia diagnosis than before the event.

**DISCUSSION:**

Incident arrhythmias are associated with accelerated cognitive decline after, but not before, the event.

**Highlights:**

- The annual rate of cognitive decline before arrhythmia diagnosis among individuals who experienced incident arrhythmias was similar to that of participants who were free of arrhythmias throughout follow‐up.
- Incident arrhythmias were not associated with an acute decrease in global cognition, memory, or executive function at the time of the event.
- The rate of decline in global cognition and memory significantly accelerated over the years after arrhythmia diagnosis.
- Preventing arrhythmias may be important in reducing long‐term cognitive decline.

## 1 BACKGROUND

Cognitive decline and dementia are common among older individuals and pose a significant public health challenge to modern societies globally.^1^ In 2019, 57.4 million people worldwide were estimated to be living with dementia, and the figure is projected to reach 152.8 million by 2050.^2^ Given the current lack of efficacious treatments for dementia, identifying modifiable risk factors for cognitive decline has become a crucial strategy to slow or prevent dementia onset. Cardiac arrhythmias, which are a group of conditions characterized by abnormalities in heart rate and/or rhythm, are expected to increase due to the aging global population.^3^ Atrial fibrillation, the most common type of cardiac arrhythmia, is a potent risk factor for stroke and has been shown to be significantly associated with an increased risk of cognitive decline and dementia.^4^, ^5^ While it is widely acknowledged that arrhythmias, particularly atrial fibrillation, have detrimental effects on cognitive function,^6^ most studies have examined only the association between arrhythmias at baseline and subsequent cognitive decline.^7^, ^8^, ^9^, ^10^ In addition, only a few longitudinal studies have explored the association between incident arrhythmias and cognitive decline, with some showing an accelerated cognitive decline associated with new‐onset arrhythmia,^11^, ^12^ while others do not show a significant association.^13^ Moreover, it remains unclear whether patients with incident arrhythmia experience faster rate of cognitive decline over the years after the event (i.e., the slope) compared to their pre‐arrhythmia rate of cognitive decline (possibly reflecting the effects of subclinical disease), after accounting for any acute cognitive decline occurring at the time of the event.

The aim of the current study is to examine the temporal trajectory of cognitive decline before and after incident arrhythmias, using repeated cognitive assessments in a large longitudinal study of older adults. We hypothesized that even in the absence of clinical stroke and coronary heart disease, participants with incident arrhythmias would experience a more rapid decline in cognitive test scores compared to their pre‐arrhythmia cognitive trajectories.

## 2 METHODS

### 2.1 Study population

The English Longitudinal Study of Ageing (ELSA) is an ongoing nationally representative, longitudinal study involving participants in England aged 50 years or older, as well as their spouses of any age.^14^ The core ELSA sample was recruited from households that participated in the 1998, 1999, or 2001 Health Survey for England (collectively referred to as wave 0). Participants were followed up biennially. We combined the longitudinal data from wave 1 (2002–2003) through wave 9 (2018–2019). The baseline survey (wave 1) included 11,391 core participants, of which 3,451 individuals were excluded from the current study for the following reasons: history of arrhythmia (*n* = 716), history of stroke (*n* = 432), history of coronary heart disease (*n* = 1,066), incomplete cognitive tests data (*n* = 271), had a confirmed diagnosis of dementia and/or Alzheimer's disease at baseline (*n* = 25), or had an incident stroke (*n* = 452) and/or coronary heart disease (*n* = 489) during follow‐up. The assessment procedures for arrhythmias, stroke, and coronary heart disease are detailed in Methods S1 in supporting information. Furthermore, we excluded participants who were lost to follow‐up during waves 2 through 9 (*n* = 1,446). Finally, the analytical sample of the current study comprised 6,494 participants with complete baseline data and at least one reassessment of cognitive function during follow‐up (between waves 2 and9). A flow chart of participant selection is presented in Figure S1 in supporting information. We focused on people without clinical stroke and coronary heart disease because the deleterious effects of these conditions on cognitive decline are already well established.^15^, ^16^, ^17^, ^18^

At each wave of ELSA, participants gave written informed consent. Ethical approval for ELSA was granted by the National Health Service Research Ethics Committee (London Multicentre Research Ethics Committee, MREC/01/2/91). This study followed the Strengthening the Reporting of Observational Studies in Epidemiology (STROBE) reporting guideline for cohort studies.

### 2.2 Cognitive assessments

Details about the cognitive measures in ELSA have been previously described.^15^, ^16^, ^19^ First, memory was assessed at each interview via the Consortium to Establish a Registry for Alzheimer's Disease immediate and delayed recall tasks.^20^ Participants were given a list of 10 unrelated words and asked to recall the list immediately and after a delay. The composite verbal memory score was calculated by summing the immediate and delayed recall scores (range from 0 to 20). Second, semantic fluency was assessed using the animal fluency task.^21^ Participants were asked to name as many animals as possible within 1 minute. The score of semantic fluency is equal to the total number of words produced, excluding repeated and non‐animal words. Third, orientation was assessed by asking four questions regarding the date (e.g., day of month, month, year, and day of week). One point was given for each correct answer, and the score of temporal orientation ranged from 0 to 4.

To allow for comparison across cognitive domains, individual cognitive scores were standardized based on the mean and standard deviation (SD) of the corresponding cognitive test at baseline. A composite global cognitive *Z* score was generated for each participant by averaging the *Z* scores of the three individual tests and then re‐standardizing using the mean and SD of the global cognitive *Z* score at baseline. Therefore, a *Z* score of 1 indicates cognitive function that is 1 SD above the mean score at baseline. To allow for comparisons of regression coefficients across different cognitive tests, the *Z* scores were used for the main analyses.

### 2.3 Assessment of incident arrhythmias

During follow‐up (wave 2 to 9), we identified participants with self‐reported doctor‐diagnosed incident arrhythmias via the following question at each wave: “abnormal heart rhythm diagnosis newly reported at this wave?” Because the accurate diagnosis date was not recorded in the ELSA, we defined the time of arrhythmia onset as the midpoint between the last visit cycle without arrhythmia and the first visit cycle with arrhythmia. This approach has been used in prior studies.^16^, ^22^, ^23^ Although the type of specific arrhythmias (e.g., atrial fibrillation/flutter, bradyarrhythmias, other supraventricular arrhythmia, and ventricular arrhythmia/cardiac arrest) is not available in the ELSA dataset, most of the incident arrhythmias are likely due to atrial fibrillation based on the demographics.^24^

### 2.4 Covariates

Structured questionnaires were administered to collect data on demographics, lifestyles, and medical history. Demographics included age, sex, education, and living alone. High education level was defined as level 3 National Vocational Qualification (NVQ3) or General Certificate of Education (GCE) A level, or above. Living alone was defined as having a household size of 1, regardless of marital status. Lifestyles included current cigarette smoking, weekly alcohol intake, physical activity, and body mass index (calculated as weight [kg]/height^2^ [m^2^]). Medical history included hypertension, diabetes, chronic lung disease, cancer, and depressive symptoms. Hypertension was defined as a systolic blood pressure of ≥ 140 mm Hg and/or a diastolic blood pressure of ≥ 90 mm Hg, self‐report of physician diagnosis of hypertension, or use of any antihypertensive medication. History of diabetes, chronic lung disease, and cancer was based on self‐reported doctor diagnoses. As body mass index and blood pressure were not measured at wave 1, we used the mean values from wave 0 and wave 2 as the estimates for wave 1. Depressive symptoms were defined as a score of ≥ 4 on the 8‐item Center for Epidemiologic Studies Depression scale.^25^

**RESEARCH IN CONTEXT**

1. **Systematic review**: A systematic review was conducted according to PROSPERO guidelines to identify studies that examined the association between arrhythmia and cognitive decline. Only a few longitudinal studies have explored the association between incident arrhythmias and cognitive decline, with some reporting accelerated cognitive decline associated with new‐onset arrhythmia, while others report no significant association. Moreover, it remains unclear whether patients with incident arrhythmias experience a faster rate of cognitive decline over the years after the event (i.e., the slope) compared to their pre‐arrhythmia rate of cognitive decline (possibly reflecting the effects of subclinical disease), after accounting for any acute cognitive decline at the time of the event.
2. **Interpretation**: Our results showed that incident arrhythmias were not associated with an acute decrease in global cognition, memory, or executive function at the time of the event compared to participants without arrhythmias. The rate of decline in global cognition and memory was significantly faster over the years after arrhythmia diagnosis compared with the decline rate pre‐arrhythmia diagnosis.
3. **Future directions**: The findings of this study suggest that preventing arrhythmias may be important for mitigating long‐term cognitive decline. Further research is needed to identify subgroups of individuals most at risk of cognitive decline following an arrhythmia event.

### 2.5 Statistical analysis

Baseline characteristics were stratified by whether an individual developed incident arrhythmias. Continuous variables are summarized as mean ± SD‐ and categorical variables as frequencies (%). Differences in baseline characteristics between those who did and did not develop incident arrhythmias were tested using Student's *t*‐test or χ^2^ test. Differences were also examined using multiple linear or logistic regression when appropriate, adjusting for age and sex.

To account for correlation among repeated cognitive measures within participants over time, we used linear mixed‐effects models to examine the association between arrhythmias and cognition changes over time, after adjusting for potential confounders.^26^ We analyzed each cognitive variable separately. We included fixed effects for intercept, time (years since baseline), incident arrhythmia (with or without arrhythmia during follow‐up), interaction between arrhythmia and time, post‐arrhythmia, time‐after‐arrhythmia, and all covariates. The basic form of the linear mixed effect model is:

$$\begin{array}{ccc} \mathrm{Cognition} & = & \beta_{0}\;(\mathrm{Intercept})+\beta_{1}\mathrm{Time}+\beta_{2}\mathrm{Arrhythmias}+\beta_{3}\mathrm{Arrhythmias}\\ & & \times \;\mathrm{Time}+\beta_{4}\mathrm{Post}-\mathrm{Arrhythmias}+\beta_{5}\mathrm{Time}-\mathrm{After}\\ & & -\;\mathrm{Arrhythmias}+\sum \beta \times \mathrm{Covariates} \end{array}$$

To allow for participant‐specific rate of cognitive change, both intercept and slope (years since baseline and years after arrhythmia) were modeled as both fixed and random effects. We are interested in the parameters $\beta_{3}$, $\beta_{4}$, and $\;\beta_{5}$. The parameter $\beta_{3}$ for the interaction term “arrhythmias×time” represented the average difference in the cognitive change rate between the arrhythmia group during the pre‐arrhythmia period and the arrhythmia‐free group across the entire follow‐up period. The variable “post‐arrhythmias” was a time‐varying incident arrhythmia variable (the value changes from 0 to 1 at the time of incident arrhythmia), and the parameter $\beta_{4}$ estimated the effect of incident arrhythmia on the acute decline in cognitive function at the time of the event. The variable “time‐after‐arrhythmia” was set to 0 for the arrhythmia‐free group and for the arrhythmia group during the pre‐arrhythmia diagnosis period. The parameter $\beta_{5}$ was used to assess whether incident arrhythmias were associated with a faster rate of cognitive decline in the years after the arrhythmia event. The detailed conceptual models are provided in Methods S1. All models were adjusted for baseline covariates, including age, sex, education, living alone, current smoking, alcohol consumption, physical activity, hypertension, diabetes, chronic lung disease, cancer, depressive symptoms, and body mass index.

After estimating the parameters for the fixed and random effects from the multivariable linear mixed model, we calculated participant‐specific (conditional) predicted values for each cognitive score over time for a 70‐year‐old woman with the average values of all covariates at baseline conditional on whether she experienced an incident arrhythmia midway through the follow‐up period (at year 8).

We conducted subgroup analyses to examine potential effect modification by baseline characteristics. For example, we divided the sample into two age categories (< 60 years and ≥ 60 years). In addition, to investigate the relationship between age of arrhythmia onset and the degree of cognitive decline before and after incident arrhythmia, we examined trends separately for participants whose arrhythmia developed before age 70 (*n* = 263) and at age ≥ 70 (*n* = 365). This classification was determined by the sample size of our arrhythmia cohort and the average age at diagnosis (71.9 years [SD 9.1]). We used the *Z* test to compare subgroup differences.^27^

Several sensitivity analyses were also conducted to examine the robustness of our findings. First, we reanalyzed the short‐term decline after arrhythmia and post‐arrhythmia diagnosis decline rate among the 628 participants who experienced an incident arrhythmia. In this population with incident arrhythmias, we fitted fixed effects only for the intercept, time, post‐arrhythmia, time‐after‐arrhythmia, and all covariates. Second, parallel to the models applied to the entire study sample, we analyzed a subset of 2,909 participants who had completed at least eight cognitive assessments. Third, we repeated the main analyses using the original cognitive scores. Fourth, we additionally adjusted for private health insurance and total wealth. Finally, to reduce non‐response bias and preserve the study sample, we used multiple imputation by chained equations to address missing cognitive scores data during follow‐up (waves 2–9) for the 7,940 participants.

Data analysis was performed between September 25, 2023, and July 22, 2024. All analyses were performed using Stata version 17.0 (StataCorp). A two‐sided *p* < 0.05 was deemed statistically significant, unless otherwise specified.

## 3 RESULTS

### 3.1 Baseline characteristics and sample size

Among the 6,494 eligible ELSA core members who were free of stroke and coronary heart disease, 58.0% were women, with a mean baseline age of 62.9 ± 9.4 years. Over a median follow‐up period of 12 years (interquartile range: 6–16 years), 628 (9.7%) incident arrhythmias were identified. The baseline characteristics of participants, stratified by incident arrhythmia status, are summarized in Table 1. After adjusting for age and sex, individuals with incident arrhythmias were more likely to have a higher education level, a greater prevalence of depressive symptoms, and a lower prevalence of smoking. The participation patterns of this study population are shown in Tables S1–S3 in supporting information. From waves 2 to 9, the cohort sizes were 6,107, 5,227, 4,631, 4,398, 4,028, 3,527, 3,091, and 2,747, and the number of participants with incident arrhythmias was 116, 66, 68, 88, 66, 73, 75, and 76, respectively. The median number of cognitive assessments was 7 (interquartile range: 3–9). The number of incident arrhythmia cases stratified by years of follow‐up is shown in Table S4 in supporting information.

**TABLE 1:** Characteristics of participants who did and did not have an incident arrhythmias during study follow‐up.

Characteristic	Total (*n* = 6494)	No incident arrhythmias (*n* = 5866)	Incident arrhythmias (*n* = 628)	*p* value^a^	Adjusted *p* value^b^
Age, years	62.9 ± 9.4	62.7 ± 9.4	64.3 ± 9.0	<0.001	NA
Women	3769 (58.0)	3421 (58.3)	348 (55.4)	0.161	NA
Education ≥ NVQ3/GCE A level^c^	2061 (31.7)	1846 (31.5)	215 (34.2)	0.159	0.034
Living alone	1486 (22.9)	1326 (22.6)	160 (25.5)	0.103	0.444
Current smoking	1145 (17.6)	1066 (18.2)	79 (12.6)	<0.001	0.003
Alcoholic drink ≥ 1 per week	4031 (62.1)	3648 (62.2)	383 (61.0)	0.555	0.667
Moderate–vigorous activity	5256 (80.9)	4753 (81.0)	503 (80.1)	0.573	0.919
Comorbidities					
Hypertension	3368 (51.9)	3017 (51.4)	351 (55.9)	0.034	0.278
Diabetes	327 (5.0)	299 (5.1)	28 (4.5)	0.487	0.305
Chronic lung disease	321 (4.9)	282 (4.8)	39 (6.2)	0.123	0.198
Cancer	363 (5.6)	321 (5.5)	42 (6.7)	0.208	0.281
Depressive symptoms	890 (13.8)	782 (13.5)	108 (17.4)	0.007	0.006
Systolic blood pressure, mm Hg	137.6 ± 18.0	137.4 ± 18.0	138.9 ± 18.4	0.068	0.537
Diastolic blood pressure, mm Hg	76.6 ± 10.4	76.7 ± 10.3	76.5 ± 11.1	0.654	0.750
Body mass index, kg/m^2^	27.7 ± 4.7	27.6 ± 4.6	28.3 ± 5.2	<0.001	<0.001
Verbal memory scores	9.9 ± 3.4	9.9 ± 3.4	10.2 ± 3.3	0.051	<0.001
Sematic fluency scores	20.1 ± 6.3	20.1 ± 6.2	20.4 ± 6.3	0.224	0.016
Temporal orientation scores	4.3 ± 2.0	4.3 ± 2.0	4.4 ± 2.0	0.037	<0.001

*Note*: Values are mean ± standard deviation or *n* (%).

Abbreviations: GCE, General Certificate of Education; NA, not applicable; NVQ3, level 3 National Vocational Qualification.

^a^ Calculated by using the Student's *t*‐test, or χ^2^ test.

^b^ Calculated by using the linear regression or logistic regression after adjustment for baseline age and sex.

^c^ NVQ3/GCE A level is equivalent to senior high school.

### 3.2 Cognitive decline before arrhythmias diagnosis

**FIGURE 1:**
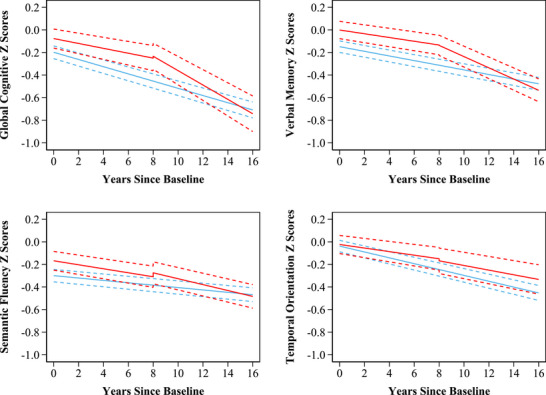
Trajectory of cognitive decline associated with incident arrhythmias. Predicted mean change in cognitive *Z* scores (standard deviation) before and after an arrhythmia at year 8. Predicted values of cognitive function were calculated for a 70‐year‐old woman with a body mass index of 28 kg/m^2^, education < NVQ3/GCE A level (equivalent to senior high school), not living alone, ≥ 1 alcoholic drink per week, moderate or vigorous activity, and hypertension, but without current smoking, diabetes, chronic lung disease, cancer, or depressive symptoms. Blue lines represent the trajectory for arrhythmia‐free participants. Red lines represent the trajectory for participants with incident arrhythmias. The dashed lines represent the 95% confidence intervals. Cognitive function was assessed biennially. GCE, General Certificate of Education; NVQ3, level 3 National Vocational Qualification

After multivariable adjustment, the annual rate of cognitive decline before arrhythmia diagnosis in individuals with incident arrhythmia was similar to that of participants who remained free of arrhythmias throughout the follow‐up period (Table 2 and Figure 1).

**TABLE 2:** Pre‐arrhythmia diagnosis annual decline in cognitive *Z* scores (SD/year), short‐term change in cognitive *Z* scores (SD), and post‐arrhythmia annual decline in cognitive *Z* scores (SD/year) after arrhythmia diagnosis.

	Pre‐arrhythmia diagnosis annual decline^a^		Short‐term change after arrhythmia diagnosis		Post‐arrhythmia diagnosis annual decline^b^	
	*β* (95% CI)^c^	*p* value	*β* (95% CI)^c^	*p* value	*β* (95% CI)^c^	*p* value
Global cognitive *Z* scores	0.010 (−0.002 to 0.023)	0.110	0.016 (−0.071 to 0.104)	0.714	−0.042 (−0.065 to −0.019)	<0.001
Verbal memory *Z* scores	0.004 (−0.005 to 0.012)	0.369	−0.003 (−0.070 to 0.064)	0.925	−0.033 (−0.047 to −0.019)	<0.001
Semantic fluency *Z* scores	−0.007 (−0.016 to 0.002)	0.135	0.035 (−0.040 to 0.109)	0.360	−0.008 (−0.023 to 0.006)	0.266
Temporal orientation *Z* scores	0.010 (−0.003 to 0.023)	0.133	−0.019 (−0.117 to 0.080)	0.713	−0.005 (−0.025 to 0.016)	0.645

Abbreviations: CI, confidence interval; SD, standard deviation.

^a^ Using participants who did not have an incident arrhythmia as the reference group.

^b^ Compared to pre‐arrhythmia diagnosis annual decline (reference).

^c^ After adjusting for baseline age, sex, education, living alone, current smoking, alcohol consumption, physical activity, hypertension, diabetes, chronic lung disease, cancer, depressive symptoms, and body mass index.

### 3.3 Short‐term change in cognitive function after arrhythmia diagnosis

As shown in Table 2 and Figure 1, participants diagnosed with arrhythmia did not experience a short‐term change in cognitive function following arrhythmia diagnosis after adjusting for pre‐ and post‐arrhythmia diagnosis cognitive decline, calendar time, and baseline covariates.

### 3.4 Mean difference between pre‐ and post‐arrhythmia diagnosis slopes

After adjusting for calendar time, short‐term change in global cognitive function, and baseline covariates, in the years after arrhythmia diagnosis, global cognitive function declined significantly faster than it did before the event (−0.042 SD/year; 95% confidence interval [CI]: −0.065 to −0.019; Table 2 and Figure 1). Similarly, the rate of decline in verbal memory (−0.033 SD/year; 95% CI: −0.047 to −0.019) was accelerated after arrhythmia diagnosis. The post‐arrhythmia diagnosis slope of semantic fluency and temporal orientation also declined faster than its pre‐arrhythmia diagnosis slope, although the difference was not statistically significant (Table 2).

### 3.5 Subgroup analyses

**FIGURE 2:**
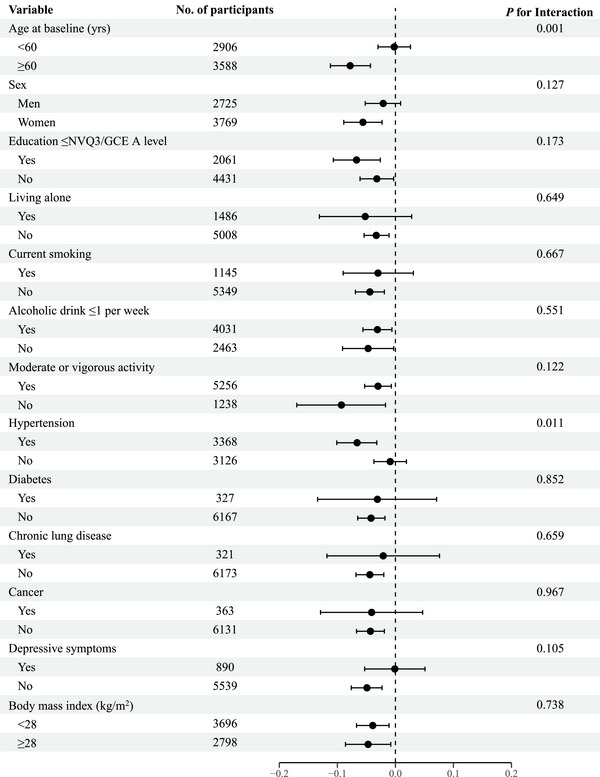
Post‐arrhythmia annual decline in global cognitive *Z* scores (standard deviation/year) after arrhythmia diagnosis, according to subgroups defined by covariates. *After adjusting for short‐term change in global cognitive *Z* scores after arrhythmias diagnosis, calendar time, baseline age, sex, education, living alone, current smoking, alcohol consumption, physical activity, hypertension, diabetes, chronic lung disease, cancer, depressive symptoms, and body mass index. GCE, General Certificate of Education; NVQ3, level 3 National Vocational Qualification; yrs, years

Further subgroup analyses showed that the post‐arrhythmia diagnosis slope was consistent across all major subgroups, except for age group (*P* for interaction = 0.001) and hypertension status (*P* for interaction = 0.011; Figure 2). After arrhythmia diagnosis,  older adults (aged ≥ 60 years) had steeper declines in global cognitive function compared with younger counterparts (−0.078 SD/year; 95% CI: −0.112 to −0.044 vs. −0.002 SD/year; 95% CI: −0.030 to 0.026). Similarly, we found that patients with hypertension showed steeper declines in global cognitive function after arrhythmia diagnosis compared with participants without baseline hypertension (−0.066 SD/year; 95% CI: −0.101 to −0.032 vs. −0.009 SD/year; 95% CI: −0.037 to 0.019).

In addition, individuals diagnosed with arrhythmia before the age of 70 years experienced a change in global cognitive function of −0.004 SD/year (95% CI: −0.030 to 0.023) over the years following the arrhythmia event. Those diagnosed at or after the age of 70 years experienced a decline of −0.119 SD/year (−0.165 to −0.073). Compared with participants who developed arrhythmias before the age of 70 years, those diagnosed later showed a significant greater decline in global cognition in the years after arrhythmia onset (*P* for interaction < 0.001; Table S5 in supporting information)

### 3.6 Sensitivity analyses

When we restricted the analyses to the 628 participants with incident arrhythmia, the post‐arrhythmia diagnosis annual declines in global cognition (−0.042 SD/year; 95% CI: −0.064 to −0.021) and verbal memory (−0.034 SD/year; 95% CI: −0.048 to −0.020) remained statistically significant after adjustment for calendar time, short‐term change in cognition, and baseline covariates (Table 3). When we restricted analyses to participants who had completed at least eight waves of cognitive measurements (*n* = 2,909), our findings were consistent with the main analyses (Table S6 in supporting information). In addition, the results of the analyses based on the original cognitive scores and additionally adjusted for private health insurance and total wealth were consistent with our main results (Tables S7 and S8 in supporting information).

**TABLE 3:** Sensitivity analysis in 628 participants with incident arrhythmia: short‐term change in cognitive *Z* scores (SD) and post‐arrhythmias annual decline in cognitive Z scores (SD/year) after arrhythmias diagnosis.

	Short‐term change after arrhythmia diagnosis		Post‐arrhythmia‐diagnosis annual decline^a^	
	*β* (95% CI)^b^	*p* value	*β* (95% CI)^b^	*p* value
Global cognitive *Z* scores	0.012 (−0.076 to 0.100)	0.788	−0.042 (−0.064 to −0.021)	<0.001
Verbal memory *Z* scores	−0.005 (−0.073 to 0.062)	0.876	−0.034 (−0.048 to −0.020)	<0.001
Semantic fluency *Z* scores	0.031 (−0.044 to 0.106)	0.419	−0.009 (−0.023 to 0.006)	0.227
Temporal orientation *Z* scores	−0.035 (−0.135 to 0.066)	0.498	−0.007 (−0.027 to 0.013)	0.500

Abbreviations: CI, confidence interval; SD, standard deviation.

^a^ Compared to pre‐arrhythmia diagnosis annual decline (reference).

^b^ After adjusting for baseline age, sex, education, living alone, current smoking, alcohol consumption, physical activity, hypertension, diabetes, chronic lung disease, cancer, depressive symptoms, and body mass index.

### 3.7 Non‐responders analysis

Of the 7,940 participants with complete baseline data, 1,446 (18.2%) were excluded from this study due to loss to follow‐up. These participants had lower baseline cognitive scores than those retained in the study. Furthermore, the excluded participants had higher percentages of covariates, including male sex, depressive symptoms, smoking, hypertension, diabetes, chronic lung disease, and cancer; they also had lower percentages of alcohol consumption, moderate‐to–vigorous intensity physical activity, and higher levels of education (Table S9 in supporting information). The results of sensitivity analyses using multiple imputed data (*n* = 7,940) were consistent with the main analysis results (Table S10 in supporting information).

## 4 DISCUSSION

In this cohort study of 6,494 middle‐aged and older adults, incident arrhythmia was associated with long‐term decline in global cognition and memory function in the years following arrhythmia diagnosis, after accounting for individuals’ cognitive changes before and acutely after the event.

Prospective longitudinal research assessing the precise effect of arrhythmias on the trajectory of cognitive decline remains limited. The compelling evidence for the link between arrhythmias and cognitive decline, as well as for the influence of arrhythmia management on cognitive function, arises from research on atrial fibrillation.^6^ Singh‐Manoux et al. used the Whitehall II study to show that, compared with participants without atrial fibrillation, those with longer exposure to atrial fibrillation (5, 10, or 15 years) experienced faster cognitive decline.^7^ Chen et al., using the Atherosclerosis Risk in Communities Neurocognitive Study over a 20 year period, demonstrated that time‐dependent atrial fibrillation was associated with a greater cognitive decline in the United States.^12^ In contrast to previous studies,^7^, ^12^ we found that compared to arrhythmia‐free individuals, those who experienced incident arrhythmia during follow‐up had a similar decline rate of cognition before its onset.

The extent of cognitive change surrounding the time of arrhythmia onset and the long‐term post‐arrhythmia cognitive trajectory, controlling for the individual's pre‐arrhythmia cognitive trajectory, has not been well described previously. The present study expands upon the exisiting literature by demonstrating that cognitive decline tends to occur gradually in the years following the onset of an arrhythmia, rather than presenting as a sudden decline immediately post‐diagnosis, as had been previously suggested. Our findings revealed no evidence of acute cognitive declines in any of the cognitive domains measured in participants diagnosed with arrhythmias. This suggests that, unlike the cognitive impairments associated with cerebrovascular events such as stroke, which are relatively sudden onset, cognitive decline associated with arrhythmias tends to accumulate gradually over time. In the case of atrial fibrillation, this may be due to subclinical cerebral emboli that progressively impair brain function and ultimately contribute to cognitive deterioration. Additionally, our findings quantified the extent of post‐arrhythmia cognitive decline. A decline of ≥ 0.5 SD from baseline has been defined as a clinically meaningful change in cognition, as reported in previous studies.^28^, ^29^ Therefore, given the significantly accelerated cognitive decline we observed in the years following arrhythmias, the 95% CIs of cognitive declines in global cognition and verbal memory over approximately 10 years would correspond to declines of 0.5 SD. In turn, monitoring for cognitive impairment in patients with arrhythmias may be warranted following arrhythmia diagnosis.

Several potential mechanisms could explain the observed associations between incident arrhythmias and long‐term cognitive decline. Incident arrhythmias can significantly impact neurological function by disrupting cerebral blood flow, oxygen delivery, and overall cardiac output.^30^ Moreover, arrhythmia survivors (e.g., those with atrial fibrillation) may experience incident cardiovascular comorbidities, such as ischemic heart disease and stroke.^31^ It is unlikely that clinically apparent stroke or coronary heart disease explains the long‐term cognitive declines, as we excluded participants with pre‐existing or newly diagnosed stroke or coronary heart disease. Still, arrhythmia survivors (e.g., atrial fibrillation) may experience covert stroke after their index arrhythmia that would contribute to subsequent cognitive decline.^32^, ^33^

Our study has several strengths. First, we used a large, nationally representative sample of middle‐aged and older adults with repeated measurements of cognitive function. Second, we accounted for pre‐arrhythmia cognitive decline and acute cognitive changes after arrhythmias to disentangle the association between arrhythmias and longitudinal cognitive decline over a 16‐year follow‐up period. However, our study also has several limitations. First, the use of self‐reported, doctor‐diagnosed incident arrhythmias may have led to misclassification, as arrhythmias encompass multiple clinically distinct entities. We did not have access to the causes of arrhythmias and therefore could not identify which type of specific arrhythmia was related to cognitive decline. Consequently, further investigation is necessary to explore the impact of specific types or severities of arrhythmias on cognitive decline. Second, although we accounted for a range of confounding variables, we could not control for the type and causes of arrhythmia, treatments, medications, and cardiac procedures, because these data were not available. Third, the ELSA sample predominantly comprised individuals of European ancestry, which may limit the generalizability of our findings to more diverse populations. Fourth, accurate information on the date of arrhythmia onset was not available. Finally, as neuroimaging data were not available, we were unable to investigate structural brain alterations and the potential mechanisms underlying the relationship between arrhythmias and long‐term cognitive decline.

In conclusion, we found that incident arrhythmias were significantly associated with faster post‐arrhythmia diagnosis cognitive decline, but not pre‐arrhythmia diagnosis or short‐term cognitive decline immediately after the event. Future studies are warranted to determine the precise mechanisms linking incident arrhythmias to cognitive decline.

## CONFLICT OF INTEREST STATEMENT

The authors report no disclosures relevant to the manuscript. Author disclosures are available in the supporting information.

## CONSENT STATEMENT

Ethical approval for ELSA was granted from the NHS Research Ethics Committee (London Multicentre Research Ethics Committee, MREC/01/2/91). All study participants provided written informed consent.

## Supporting information



Supporting Information

Supporting Information
