# Supplementary material for: Trajectory of cognitive decline before and after incident arrhythmias in older adults: A 16‐year population‐based longitudinal cohort study
Source: Alzheimers Dement. 2025 May 19;21(5):e70260. doi: 10.1002/alz.70260 (PMC12086794; doi:10.1002/alz.70260)
Supplement: Supplementary file 1 — Supporting Information [file ALZ-21-e70260-s001.docx]

**Supplementary Online Content**

**Method S1**

**Assessment of arrhythmias**

At baseline (wave 1), participants were identified as having a history of arrhythmias based on their response to the question: Has a doctor ever told you that you [have/have had] an abnormal heart rhythm? During follow-up (wave 2 to wave 9), incident arrhythmias cases were identified at each wave by asking: “abnormal heart rhythm diagnosis newly reported at this wave.

**Assessment of stroke**

At baseline (wave 1), participants were identified as having a history of stroke based on their response to the question: Has a doctor ever told you that you [have/have had] a stroke (cerebral vascular disease)? During follow-up (wave 2 to wave 9), incident stroke cases were identified at each wave by asking: “stroke diagnosis newly reported at this wave.

**Assessment of coronary heart disease**

Participants were classiﬁed as having coronary heart disease (CHD) if they had been diagnosed with myocardial infarction and/or angina. At baseline (wave 1), we identified participants with a history of CHD through this question: Has a doctor ever told you that you [have/have had] a heart attack (including myocardial infarction or coronary thrombosis) or angina. During follow-up (wave 2 to wave 9), we identified participants with incident CHD via the following question at each wave: “heart attack (myocardial infarction or coronary thrombosis) diagnosis newly reported” or “angina diagnosis newly reported”.

**Statistical analysis**

The conceptual model of our study is shown as follows:


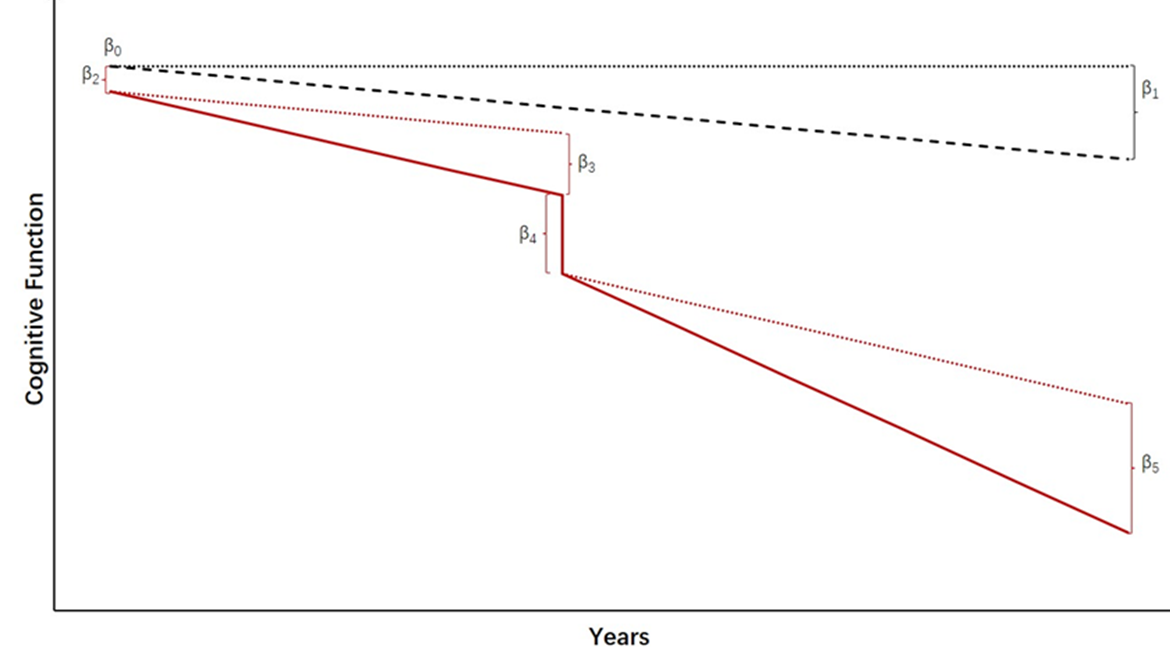


The black dashed line represents the possible trajectory of the control group without incident stroke. The cognitive trajectories of the arrhythmias group (red lines) consisted of the trajectories before arrhythmias, an acute decline at the time of arrhythmias, and an accelerated decline after arrhythmias.

The parameter $\text{β}_{\text{1}}$reﬂected the average rate of cognitive change for the entire arrhythmias-free group. The variable “arrhythmias” was coded 1 for individual who experienced incident arrhythmias during the follow-up and 0 for those who did not. The parameter $\text{β}_{\text{2}}$reﬂect the baseline cognition diﬀerence between the arrhythmias group and the arrhythmias-free group. The parameter $\text{β}_{\text{3}}$for the interaction term “$\text{arrhythmias×time}$” reﬂected the average diﬀerence in the cognitive change rate between the arrhythmias group during the pre-arrhythmias period and the arrhythmias-free group during the whole follow-up period. The variable “post-arrhythmias” was a time-varying incident arrhythmias variable (the value changes from 0 to 1 at the time of incident arrhythmia) and the parameter $\text{β}_{\text{4}}$estimated the effect of incident arrhythmias on the acute decline in cognitive function at the time of the event. The variable “time-after-arrhythmia” was set to 0 for the arrhythmias-free group and for the arrhythmias group during the pre-arrhythmia diagnosis period. The parameter $\text{β}_{\text{5}}$could assess whether incident arrhythmias were associated with a faster rate of cognitive decline in the years following the arrhythmias event.


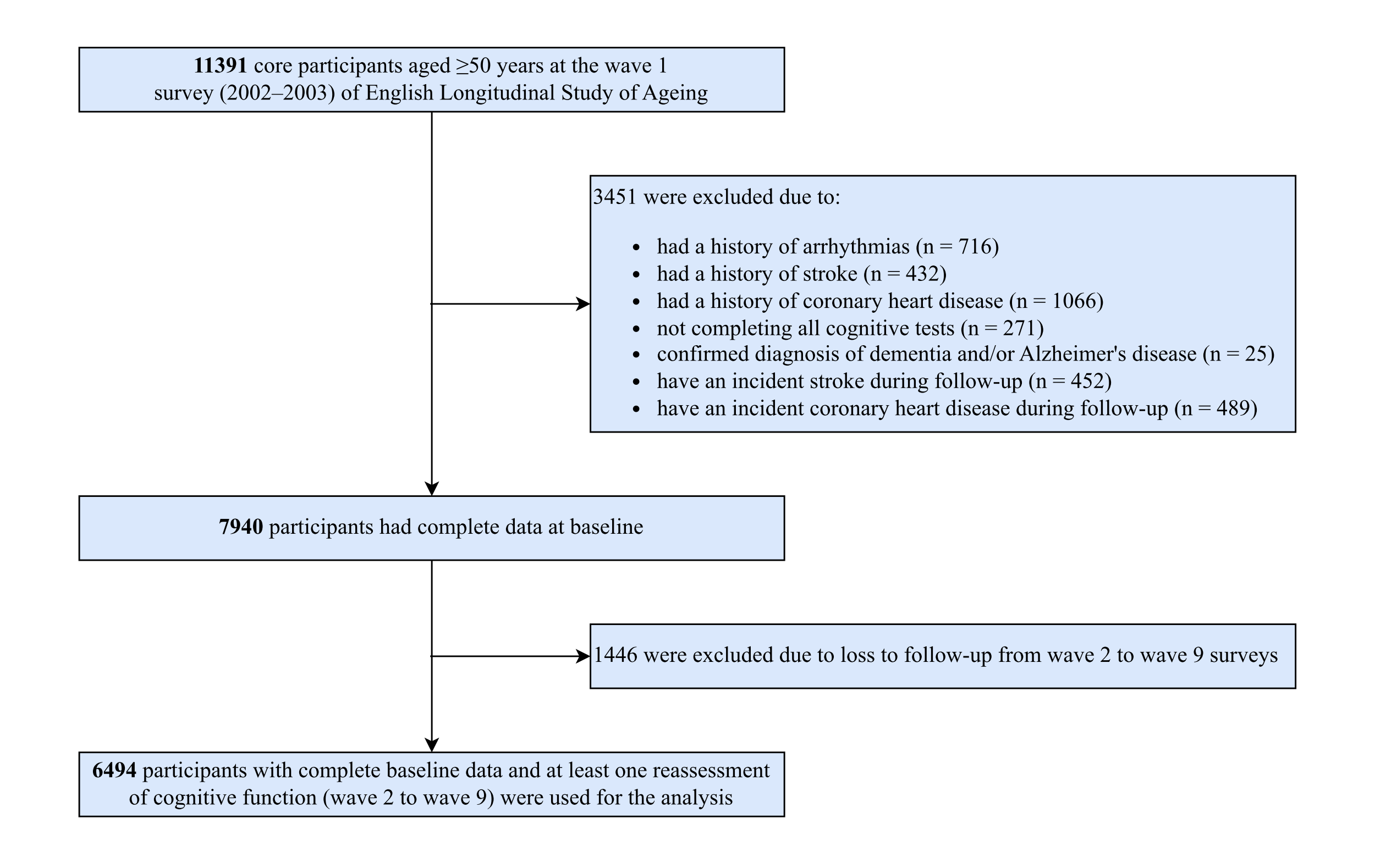


**Figure S1. Study Participants Selection from the English Longitudinal Study of Ageing**

| **Table S1. The numbers of participants in the incident arrhythmias group and in the non-arrhythmias group at waves 1 to 9** | | | |
| --- | --- | --- | --- |
|  | **Numbers of participants in two groups** | |  |
| **Waves** | **Non-arrhythmias group (%**^*^**)** | **Incident arrhythmias groups (%**^*^**)** | **Total (%**^*^**)** |
| At wave 1 (2002-2003) | 5866 (100.0) | 628 (100.0) | 6494 (100.0) |
| At wave 2 (2004-2005) | 5506 (93.9) | 601 (95.7) | 6107 (94.0) |
| At wave 3 (2006-2007) | 4658 (79.4) | 569 (90.6) | 5227 (80.5) |
| At wave 4 (2008-2009) | 4077 (69.5) | 554 (88.2) | 4631 (71.3) |
| At wave 5 (2010-2011) | 3841 (65.5) | 557 (88.7) | 4398 (67.7) |
| At wave 6 (2012-2013) | 3516 (59.9) | 512 (81.5) | 4028 (62.0) |
| At wave 7 (2014-2015) | 3060 (52.2) | 467 (74.4) | 3527 (54.3) |
| At wave 8 (2016-2017) | 2675 (45.6) | 416 (66.2) | 3091 (47.6) |
| At wave 9 (2018-2019) | 2390 (40.7) | 357 (56.8) | 2747 (42.3) |
| *Attendance percentages. | | | |

| **Table S2. The numbers of participants in the incident arrhythmias group and in the non-arrhythmias group, by the total number of attending waves** | | | |
| --- | --- | --- | --- |
|  | **Numbers of participants in two groups** | |  |
| **Total number of**  **attending waves** | **Non-arrhythmias group (n = 5866)** | **Incident arrhythmias groups (n = 628)** | **Total**  **(n = 6494)** |
| 9 waves (%) | 1969 (33.6) | 301 (47.9) | 2270 (35.0) |
| 8 waves (%) | 547 (9.3) | 92 (14.6) | 639 (9.8) |
| 7 waves (%) | 411 (7.0) | 68 (10.8) | 479 (7.4) |
| 6 waves (%) | 459 (7.8) | 48 (7.6) | 507 (7.8) |
| 5 waves (%) | 418 (7.1) | 44 (7.0) | 462 (7.1) |
| 4 waves (%) | 485 (8.3) | 24 (3.8) | 509 (7.8) |
| 3 waves (%) | 677 (11.5) | 34 (5.4) | 711 (10.9) |
| 2 waves (%) | 900 (15.3) | 17 (2.7) | 917 (14.1) |

| **Table S3. The distribution of attending waves among 628 participants with incident arrhythmias** | | | | | | | | | | |
| --- | --- | --- | --- | --- | --- | --- | --- | --- | --- | --- |
| **Self-reported incident arrhythmias at different waves** | **Total** | **Wave 1 (%)** | **Wave 2 (%)** | **Wave 3 (%)** | **Wave 4 (%)** | **Wave 5 (%)** | **Wave 6 (%)** | **Wave 7 (%)** | **Wave 8 (%)** | **Wave 9 (%)** |
| At wave 2 | 116 | 116 (100.0) | 116 (100.0) | 99 (85.3) | 83 (71.6) | 76 (65.5) | 67 (57.8) | 57 (49.1) | 51 (44.0) | 44 (37.9) |
| At wave 3 | 66 | 66 (100.0) | 61 (92.4) | 66 (100.0) | 54 (81.8) | 52 (78.8) | 43 (65.2) | 40 (60.6) | 30 (45.5) | 27 (40.9) |
| At wave 4 | 68 | 68 (100.0) | 63 (92.6) | 59 (86.8) | 68 (100.0) | 59 (86.8) | 47 (69.1) | 39 (57.4) | 30 (44.1) | 26 (38.2) |
| At wave 5 | 88 | 88 (100.0) | 85 (96.6) | 77 (87.5) | 74 (84.1) | 88 (100.0) | 70 (79.5) | 57 (64.8) | 49 (55.7) | 36 (40.9) |
| At wave 6 | 66 | 66 (100.0) | 61 (92.4) | 57 (86.4) | 61 (92.4) | 63 (95.5) | 66 (100.0) | 57 (86.4) | 54 (81.8) | 37 (56.1) |
| At wave 7 | 73 | 73 (100.0) | 69 (94.5) | 67 (91.8) | 69 (94.5) | 71 (97.3) | 72 (98.6) | 73 (100.0) | 56 (76.7) | 45 (61.6) |
| At wave 8 | 75 | 75 (100.0) | 73 (97.3) | 72 (96.0) | 73 (97.3) | 74 (98.7) | 73 (97.3) | 73 (97.3) | 75 (100.0) | 66 (88.0) |
| At wave 9 | 76 | 76 (100.0) | 73 (96.1) | 72 (94.7) | 72 (94.7) | 74 (97.4) | 74 (97.4) | 71 (93.4) | 71 (93.4) | 76 (100.0) |

| **Table S4**. **The number of incident arrhythmia cases stratified by years of follow-up** | | |
| --- | --- | --- |
| **Follow-up time** | **Number of incident cases** | **Percent (%)** |
| 0–2 | 116 | 18.47 |
| 2-4 | 66 | 10.51 |
| 4-6 | 68 | 10.83 |
| 6-8 | 88 | 14.01 |
| 8-10 | 66 | 10.51 |
| 10-12 | 73 | 11.62 |
| 12-14 | 75 | 11.94 |
| 14-16 | 76 | 12.10 |
| Total | 628 | 100.00 |

| **Table S5. Subgroup analysis according to age at arrhythmias onset: short-term change in global cognitive Z scores (SD) and post-arrhythmias annual decline in global cognitive Z scores (SD/year) after arrhythmias diagnosis** | | | | | | |
| --- | --- | --- | --- | --- | --- | --- |
|  | **Age at Onset < 70 Years (n = 263)** | |  | **Age at Onset ≥ 70 Years (n = 365)** | | ***P* for Interaction** |
|  | ***β* (95% CI)**‡ | ***P* value** |  | ***β* (95% CI)**‡ | ***P* value** |  |
| Pre-Arrhythmias-Diagnosis Annual Decline* | 0.010 (−0.011 to 0.031) | 0.347 |  | 0.011 (−0.005 to 0.026) | 0.182 | 0.968 |
| Short-Term Change After Arrhythmias Diagnosis | 0.115 (−0.009 to 0.239) | 0.069 |  | 0.030 (−0.099 to 0.158) | 0.651 | 0.348 |
| Post-Arrhythmia-Diagnosis  Annual Decline† | −0.004 (−0.030 to 0.023) | 0.796 |  | −0.119 (−0.165 to −0.073) | <0.001 | <0.001 |
| *Using participants who did not have an incident arrhythmia as the reference group (n = 5866). | | | | | | |
| **†**Compared with pre-arrhythmias-diagnosis annual decline (reference). | | | | | | |
| ‡After adjusting for baseline age, sex, education, living alone, current smoking, alcohol consumption, physical activity, hypertension, diabetes, chronic lung disease, cancer, depression symptoms, and body mass index. | | | | | | |

| **Table S6. Sensitivity analysis in a subgroup of participants who have at least 8 waves of complete data (n = 2909): pre-arrhythmias-diagnosis annual decline in cognitive z scores (SD/year), short-term change in cognitive z scores (SD), and post-arrhythmias annual decline in cognitive z scores (SD/year) after arrhythmias diagnosis** | | | | | | | | |
| --- | --- | --- | --- | --- | --- | --- | --- | --- |
|  | **Pre-Arrhythmias-Diagnosis Annual Decline*** | |  | **Short-Term Change After Arrhythmias Diagnosis** | |  | **Post-Arrhythmia-Diagnosis**  **Annual Decline†** | |
|  | ***β* (95% CI)**‡ | ***P* value** |  | ***β* (95% CI)**‡ | ***P* value** |  | ***β* (95% CI)**‡ | ***P* value** |
| Global cognitive Z scores | −0.004 (−0.016 to 0.008) | 0.559 |  | 0.061 (−0.035 to 0.157) | 0.216 |  | −0.030 (−0.051 to −0.008) | 0.006 |
| Verbal memory Z scores | 0.000 (−0.009 to 0.009) | 0.949 |  | 0.034 (−0.044 to 0.112) | 0.391 |  | −0.034 (−0.050 to −0.019) | <0.001 |
| Semantic ﬂuency Z scores | −0.013 (−0.023 to −0.003) | 0.014 |  | 0.064 (−0.026 to 0.155) | 0.163 |  | −0.006 (−0.023 to 0.010) | 0.435 |
| Temporal orientation Z scores | 0.002 (−0.011 to 0.014) | 0.774 |  | 0.013 (−0.093 to 0.118) | 0.812 |  | −0.008 (−0.029 to 0.013) | 0.445 |
| *Using participants who did not have an incident arrhythmia as the reference group. | | | | | | | | |
| **†**Compared with pre-arrhythmias-diagnosis annual decline (reference). | | | | | | | | |
| ‡After adjusting for baseline age, sex, education, living alone, current smoking, alcohol consumption, physical activity, hypertension, diabetes, chronic lung disease, cancer, depression symptoms, and body mass index. | | | | | | | | |

| **Table S7. Pre-arrhythmias-diagnosis annual decline in** **original cognitive scores (points/year), short-term change in original cognitive scores (points), and post-arrhythmias annual decline in original cognitive scores (points/year) after arrhythmias diagnosis** | | | | | | | | |
| --- | --- | --- | --- | --- | --- | --- | --- | --- |
|  | **Pre-Arrhythmias-Diagnosis**  **Annual Decline*** | |  | **Short-Term Change After Arrhythmias Diagnosis** | |  | **Post-Arrhythmia-Diagnosis**  **Annual Decline†** | |
|  | ***β* (95% CI)**‡ | ***P* value** |  | ***β* (95% CI)**‡ | ***P* value** |  | ***β* (95% CI)**‡ | ***P* value** |
| Verbal memory Z scores | 0.013 (−0.016 to 0.042) | 0.369 |  | −0.011 (−0.239 to 0.217) | 0.925 |  | −0.112 (−0.160 to −0.065) | <0.001 |
| Semantic ﬂuency Z scores | −0.045 (−0.103 to 0.014) | 0.135 |  | 0.218 (−0.249 to 0.685) | 0.360 |  | −0.053 (−0.146 to 0.040) | 0.266 |
| Temporal orientation Z scores | 0.005 (−0.002 to 0.011) | 0.133 |  | −0.009 (−0.057 to 0.039) | 0.713 |  | −0.002 (−0.012 to 0.008) | 0.645 |
| *Using participants who did not have an incident arrhythmia as the reference group. | | | | | | | | |
| **†**Compared with pre-arrhythmias-diagnosis annual decline (reference). | | | | | | | | |
| ‡After adjusting for baseline age, sex, education, living alone, current smoking, alcohol consumption, physical activity, hypertension, diabetes, chronic lung disease, cancer, depression symptoms, and body mass index. | | | | | | | | |

| **Table S8. Sensitivity analysis by additionally adjusting for private health insurance and total wealth: pre-arrhythmias-diagnosis annual decline in cognitive Z Scores (SD/year), short-term change in cognitive Z scores (SD), and post-arrhythmias annual decline in cognitive Z scores (SD/year) after arrhythmias diagnosis** | | | | | | | | |
| --- | --- | --- | --- | --- | --- | --- | --- | --- |
|  | **Pre-Arrhythmias-Diagnosis Annual Decline*** | |  | **Short-Term Change After Arrhythmias Diagnosis** | |  | **Post-Arrhythmia-Diagnosis**  **Annual Decline†** | |
|  | ***β* (95% CI)**‡ | ***P* value** |  | ***β* (95% CI)**‡ | ***P* value** |  | ***β* (95% CI)**‡ | ***P* value** |
| Global cognitive Z scores | 0.010 (−0.003 to 0.023) | 0.122 |  | 0.019 (−0.069 to 0.107) | 0.678 |  | −0.043 (−0.067 to −0.020) | <0.001 |
| Verbal memory Z scores | 0.003 (−0.005 to 0.012) | 0.472 |  | −0.002 (−0.069 to 0.066) | 0.965 |  | −0.033 (−0.047 to −0.018) | <0.001 |
| Semantic ﬂuency Z scores | −0.007 (−0.017 to 0.002) | 0.138 |  | 0.032 (−0.044 to 0.107) | 0.411 |  | −0.008 (−0.023 to 0.007) | 0.280 |
| Temporal orientation Z scores | 0.011 (−0.003 to 0.024) | 0.120 |  | −0.017 (−0.118 to 0.083) | 0.732 |  | −0.006 (−0.027 to 0.015) | 0.557 |
| *Using participants who did not have an incident arrhythmia as the reference group. | | | | | | | | |
| **†**Compared with pre-arrhythmias-diagnosis annual decline (reference). | | | | | | | | |
| ‡After adjusting for baseline age, sex, education, living alone, current smoking, alcohol consumption, physical activity, hypertension, diabetes, chronic lung disease, cancer, depression symptoms, body mass index, private health insurance, and total wealth. | | | | | | | | |

| **Table S9. Comparison of baseline characteristics between participants included (n=6494) and excluded due to loss to follow-up (n=1446)** | | | |
| --- | --- | --- | --- |
| **Characteristic** | **Included (n=6494)** | **Loss to follow-up**  **(n=1446)** | ***P* for**  **difference*** |
| Age, yrs | 62.9 ± 9.4 | 66.6 ± 11.4 | <0.001 |
| Women | 3769 (58.0) | 783 (54.1) | 0.007 |
| Education ≥ NVQ3/GCE A level‡ | 2061 (31.7) | 298 (20.6) | <0.001 |
| Living alone | 1486 (22.9) | 397 (27.5) | <0.001 |
| Current smoking | 1145 (17.6) | 298 (20.6) | 0.008 |
| Alcoholic drink ≥1 per week | 4031 (62.1) | 782 (54.2) | <0.001 |
| Moderate-vigorous activity | 5256 (80.9) | 976 (67.5) | <0.001 |
| Hypertension | 3368 (51.9) | 759 (52.5) | 0.67 |
| Diabetes | 327 (5.0) | 114 (7.9) | <0.001 |
| Chronic lung disease | 321 (4.9) | 110 (7.6) | <0.001 |
| Cancer | 363 (5.6) | 108 (7.5) | 0.006 |
| Depressive symptoms | 890 (13.8) | 251 (17.6) | <0.001 |
| Systolic blood pressure, mm Hg | 137.6 ± 18.0 | 144.6 ± 20.9 | <0.001 |
| Diastolic blood pressure, mm Hg | 76.6 ± 10.4 | 78.1 ± 12.4 | <0.001 |
| Body mass index, kg/m^2^ | 27.7 ± 4.7 | 27.5 ± 4.9 | 0.22 |
| Verbal memory scores | 9.9 ± 3.4 | 8.4 ± 3.8 | <0.001 |
| Sematic ﬂuency scores | 20.1 ± 6.3 | 17.2 ± 6.5 | <0.001 |
| Temporal orientation scores | 4.3 ± 2.0 | 3.5 ± 2.2 | <0.001 |
| The results are presented as mean ± SD or n (%).  ^*^Calculated by using a t test or chi-square test.  ‡NVQ3/GCE A level is equivalent to senior high school. | | | |

| **Table S10. Sensitivity Analysis by multiple imputation to address missingness due to nonresponse bias: pre-arrhythmias-diagnosis annual decline in cognitive Z scores (SD/year), short-term change in cognitive Z scores (SD), and post-arrhythmias annual decline in cognitive Z scores (SD/year) After arrhythmias diagnosis** | | | | | | | | |
| --- | --- | --- | --- | --- | --- | --- | --- | --- |
|  | **Pre-Arrhythmias-Diagnosis Annual Decline*** | |  | **Short-Term Change After Arrhythmias Diagnosis** | |  | **Post-Arrhythmia-Diagnosis**  **Annual Decline†** | |
|  | ***β* (95% CI)**‡ | ***P* value** |  | ***β* (95% CI)**‡ | ***P* value** |  | ***β* (95% CI)**‡ | ***P* value** |
| Global cognitive Z scores | 0.007 (−0.004 to 0.018) | 0.232 |  | 0.002 (−0.077 to 0.080) | 0.962 |  | −0.036 (−0.056 to −0.016) | <0.001 |
| Verbal memory Z scores | 0.001 (−0.007 to 0.009) | 0.764 |  | −0.007 (−0.071 to 0.056) | 0.823 |  | −0.029 (−0.042 to −0.016) | <0.001 |
| Semantic ﬂuency Z scores | −0.009 (−0.018 to − 0.0002) | 0.046 |  | 0.031 (−0.041 to 0.102) | 0.400 |  | −0.007 (−0.041 to 0.102) | 0.303 |
| Temporal orientation Z scores | 0.007 (−0.005 to 0.019) | 0.248 |  | −0.030 (−0.119 to 0.059) | 0.511 |  | −0.003 (−0.021 to 0.016) | 0.772 |
| *Using participants who did not have an incident arrhythmia as the reference group. | | | | | | | | |
| **†**Compared with pre-arrhythmias-diagnosis annual decline (reference). | | | | | | | | |
| ‡After adjusting for baseline age, sex, education, living alone, current smoking, alcohol consumption, physical activity, hypertension, diabetes, chronic lung disease, cancer, depression symptoms, and body mass index. | | | | | | | | |
